# Supplementary material for: Vitamin D Modified DSS-Induced Colitis in Mice via STING Signaling Pathway
Source: Biology (Basel). 2025 Jun 18;14(6):715. doi: 10.3390/biology14060715 (PMC12190092; doi:10.3390/biology14060715)
Supplement: Supplementary file 1 [file biology-14-00715-s001.zip › Supplementary Materials S1: 16s-report of gut microbiota/Groups/diff_analysis/RF/GMM_Groups.RFimportance.pdf]

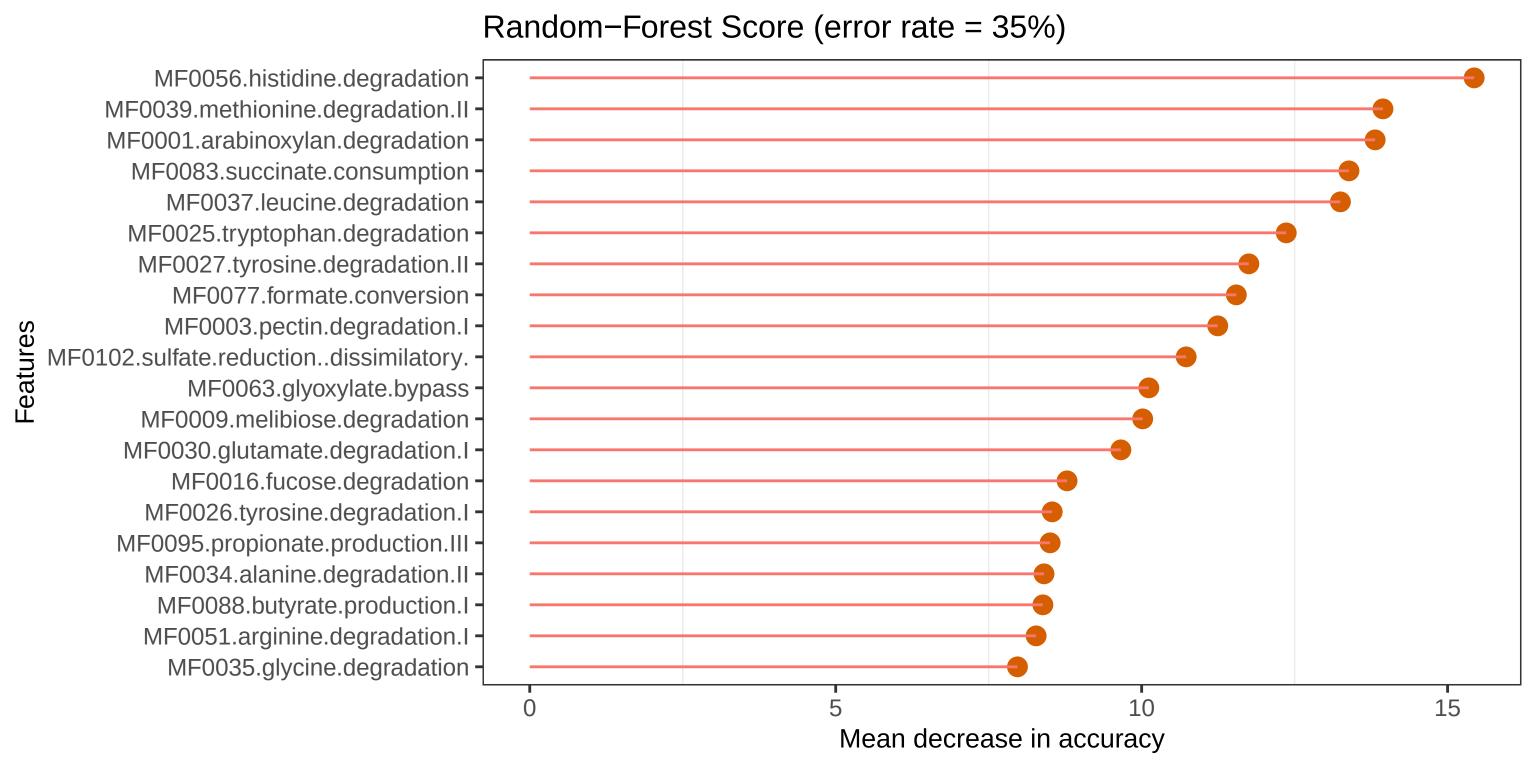
Random−Forest Score (error rate = 35%)
Features
MF0056.histidine.degradation
MF0039.methionine.degradation.II
MF0001.arabinoxylan.degradation
MF0083.succinate.consumption
MF0037.leucine.degradation
MF0025.tryptophan.degradation
MF0027.tyrosine.degradation.II
MF0077.formate.conversion
MF0003.pectin.degradation.I
MF0102.sulfate.reduction..dissimilatory.
MF0063.glyoxylate.bypass
MF0009.melibiose.degradation
MF0030.glutamate.degradation.I
MF0016.fucose.degradation
MF0026.tyrosine.degradation.I
MF0095.propionate.production.III
MF0034.alanine.degradation.II
MF0088.butyrate.production.I
MF0051.arginine.degradation.I
MF0035.glycine.degradation
0
5
10
15
Mean decrease in accuracy
